# Maturity-Dependent Volatile Flavor Profiling of Baked Potatoes via HS-SPME-GC-MS, Multivariate Statistical Analysis, and Computational Modeling

**DOI:** 10.3390/foods15091468

**Published:** 2026-04-22

**Authors:** Hong Jiang, Jingshan Guo, Zhigang Han, Jianfei Xu, Fankui Zeng

**Affiliations:** 1 Lanzhou Institute of Chemical Physics, Chinese Academy of Sciences (CAS), Lanzhou 730000, China; 2 Inner Mongolia Academy of Agricultural and Animal Husbandry Sciences, Hohhot 010020, China; 3 State Key Laboratory of Vegetable Biobreeding, Key Laboratory of Biology and Genetic Improvement of Tuber and Root Crop of Ministry of Agriculture and Rural Affairs, Institute of Vegetables and Flowers, Chinese Academy of Agricultural Sciences, Beijing 100081, China

**Keywords:** potatoes, maturity, baking, volatile compounds, HS-SPME-GC-MS

## Abstract

This study explored the flavor profiling of baked potatoes, with a focus on how maturity affects the volatile flavor. By using HS-SPME-GC-MS, sensory evaluation, multivariate statistical analysis and computational modeling, a total of 99 volatile compounds were finally identified. Multivariate statistical analysis yielded 36 different important compounds (VIP > 1, *p* < 0.05). Subsequently, combined with relative odor activity value (ROAV), four key compounds including 2-ethyl-3,5-dimethylpyrazine, 2,6-diethylpyrazine, ethyl acetate and benzeneacetaldehyde were identified as potential indicators of baked potatoes with different maturities. Further, molecular docking analysis revealed the interactions between key pyrazine compounds and human olfactory receptors OR5K1 through hydrogen bonds and other interactions. These findings provide new insights into the relationship between potato maturity and flavor differences, and also lays a foundation for in-depth exploration into flavor identification and perception.

## 1. Introduction

As the world’s fourth most important staple crop after maize, rice and wheat, the global potato production had exceeded 380 million metric tons in 2017 [1]. Due to its rich nutritional components such as carbohydrates, protein, fiber, minerals and other bioactive compounds, potatoes are widely considered as a valuable food resource [2]. China is currently the largest potato-producer, with an output of 93.5 million tons in 2023 [3]. Among them, approximately 50% of harvested potatoes in China are consumed as fresh potatoes or used for food processing. In the process of direct consumption, the flavor and texture are particularly important to consumer choice. With increasing living standards, consumer demand is shifting towards improved tuber quality, not only in terms of nutrition, but also in terms of flavor [4]. These preferences are also emphasized in breeding efforts, such as root, tuber, and banana (RTB) breeding programs, which aim to integrate end-user traits into varietal development [5].

Baked potatoes, a popular snack, are appreciated for their unique taste and convenience [6]. Additionally, baking enhances flavor through the generation of numerous volatile compounds, which significantly improve consumer acceptance [7]. More than 390 kinds of compounds have been reported to have been identified in baked potatoes, including aldehydes, alcohols, ketones, acids, esters, hydrocarbons, amines, furans and sulfur compounds [8]. The formation of these pleasant flavors is primarily driven by thermal and enzymatic reactions among various precursors, including fatty acids, amino acids, and sugars. The main reactions include the Maillard reaction, Strecker amino acid degradation, lipid degradation and other sugar degradation reactions [9,10]. However, the formation of flavor compounds in the baked potatoes is affected by various factors, such as cultivars, cultivation, storage conditions and tuber maturity. Although numerous studies have examined the effects of cultivar, agronomic conditions, and storage, limited research has focused specifically on the impact of maturity on volatile profiles.

Potato maturity, typically expressed in days from emergence to physiological maturity, is a crucial trait in breeding programs [11]. Based on growth duration, potato cultivars are generally classified as early, mid-, and late-maturing [12]. Early maturing cultivars are characterized by rapid development and a shorter growth period. Under favorable cultivation conditions, their yield may even outperform later cultivars, while late-maturing cultivars can accumulate more nutrients due to sufficient photosynthesis during their long growth cycle, resulting in better flavor and storability. Therefore, selecting cultivars with appropriate maturity for specific environmental conditions can thus optimize both yield and resource utilization [13,14]. In China, large-scale potato production occurs across four main agro-ecological zones: the northern single-planting region, the central double-planting region, the southern winter-planting region, and the southwestern mixed-planting region. In each cultivation region, appropriate potato varieties with different maturities must be selected based on local climatic and geographical conditions [15].

It has been reported that potato maturity can affect the storage quality (e.g., weight loss, sugar content and secondary metabolites) and processing quality [16], with late-maturing cultivars generally exhibiting superior processing properties compared to early maturing ones [17]. Consistent with these findings, the accumulation of proximate composition and bioactive compounds (e.g., anthocyanins, flavonoid and polyphenols) are also maturity dependent [18]. Early maturing cultivars tend to accumulate higher levels of certain biologically active substances, i.e., chlorogenic acid [19], while late-maturing cultivars easily accumulate higher levels of total and free amino acids [20]. Similarly, phenolic acid and flavonoid contents were reported to be higher in early maturing cultivars, while alkaloid contents increased in late-maturing ones [21]. Metabolomics profiling further supports the metabolite differences between early and late-maturing varieties [12].

However, the differences in processing flavors among potato cultivars with different maturities, as well as the factors contributing to these differences, have not been systematically elucidated. Therefore, this study aims to investigate the differences in flavor among early, mid-, and late-maturing potato cultivars grown in Inner Mongolia. In this research, the chemical composition including total starch, amylose, dry matter, and total sugar was first investigated, then the volatile flavors of baked potatoes were identified using headspace solid-phase microextraction-gas chromatography-mass spectrometry (HS-SPME-GC-MS) together with sensory evaluation. Then, multivariate statistical analysis was performed to screen the key volatiles that contributed the most to the overall aroma. Furthermore, computational modeling was also carried out in order to reveal the interactions between symbolic pyrazines in baked potatoes and human olfactory receptors. This research provides foundational insights into the differences in volatile formation in potatoes of varying maturity and offers a theoretical basis for flavor-oriented potato breeding.

## 2. Materials and Methods

### 2.1. Materials Preparation

Six cultivars of three different maturities were harvested in October 2024 from Wuchuan County, Hohhot City, and the Inner Mongolia Autonomous Region. These cultivars included early maturing cultivars ‘Zhongshuzao No. 35’ and ‘Huashu No. 16’, middle-maturing cultivars ‘Yanshu No. 13’ and ‘Xueyu No. 6’, and late-maturing cultivars ‘Dingshu No. 6’ and ‘Longshu No. 12’. All of these cultivars were cultivated under uniform agricultural practice at the same experimental station (40°47′ N, 110°31′ E) in Wuchuan County. The growth cycle for early maturing, middle-maturing and late-maturing cultivars are 68–70 days, 80–90 days and 100–120 days, respectively.

After harvest, undamaged, disease- and pest-free, non-greening tubers were selected, transported to the laboratory and prepared for further analysis. The selected potato tubers of each maturity were washed, air-dried and divided into two groups: raw and baked. For baking, unpeeled tubers were halved and baked at 200 °C for 1.0 h in a conditional oven. After cooling to room temperature, the baked samples were manually peeled, freeze-dried, ground into powder, and stored for chemical analyses. Raw tubers were diced into small pieces and immediately frozen in liquid nitrogen for use.

### 2.2. Chemical Components of Fresh and Baked Potatoes

#### 2.2.1. Total Starch and Amylose Contents

Total starch assay was performed using a commercial kit (AKSU015C, Beijing Boxbio Science & Technology, Beijing, China) according to the manufacturer’s instructions. About 0.05 g of sample with 1 mL eluent was ground in a mortar, incubated in a water bath at 80 °C for 30 min, and centrifuged at 8400 rpm at room temperature for 10 min. The obtained precipitate was kept and 500 μL of distilled water was added to it, mixed thoroughly, and then gelatinized in a water bath at 95 °C for 15 min. After cooling to room temperature, 1 mL of extraction solution was added to the gelatinized samples, mixed, and extracted for 15 min. Then, it was centrifuged again at 8400 rpm for 10 min and the supernatant was taken as the sample for analysis. Finally, the anthrone was added and reacted with acid hydrolysis product 5-hydroxymethylfurfural, leading to the generation of furfural derivatives. The system was heated at 95 °C for 10 min to terminate the reaction, and then the absorbance at 510 nm was measured. The starch content was calculated according to the standard curve of glucose.

Amylose assay was performed using a commercial kit (AKSU016C, Beijing Boxbio Science & Technology, Beijing, China) according to the manufacturer’s instructions. The sample was first dried and ground thoroughly, then 0.02 g of the dried sample was mixed with 1 mL of extraction solution, homogenized, extracted in a water bath at 80 °C for 30 min and centrifuged at 4400 rpm for 5 min, after which it was cooled to room temperature. The precipitate was kept, added with correspondent regent, and then centrifuged at 4400 rpm for 5 min once again. The obtained precipitate was mixed with the provided regent in the kit, incubated at 90 °C for 10 min, and then centrifuged as above. After that, a mixture containing 100 μL of supernatant, 20 μL of regent VI and regent VII, and 860 μL of distilled water were mixed and recorded at 550 nm and 485 nm. The amylose content was calculated from the determined standard curve of the standards.

#### 2.2.2. The Dry Matter and Total Sugar Contents

The determination of the dry matter contents of fresh potatoes and baked potatoes was investigated as described by Niu et al. [22]. Samples of 25 g were first fixed in an oven at 100 °C for 20 min, followed by drying under a constant weight at 80 °C. The dry matter content was calculated by dividing the constant weight by the fresh weight and expressed as a percentage. The total sugar assay was performed following a specific protocol detailed in the kit (AKSU016C, Beijing Boxbio Science & Technology, Beijing, China) manual. A 0.1 g sample was mixed with 1 mL of extraction solution A and 1.5 mL of distilled water, homogenized, and subjected to a water bath at 30 °C for 30 min. After cooling to room temperature, 1 mL of extraction solution B was added, after which the volume was then adjusted to 10 mL, and then centrifuged at 8500 rpm for 10 min. The supernatant was used for determination. The total sugars were decomposed into reducing sugars with free aldehyde and ketone groups through acid hydrolysis. Finally, 3, 5-dinitrosalicylic acid was added to the supernatant, yielding brownish-red amino compounds, the characteristic absorption peaks of which can be measured at 540 nm. The total sugar content was calculated according to the standard curve.

### 2.3. Sensory Evaluation

Sensory evaluation of baked potatoes was performed in a sensory laboratory at the Lanzhou Institute of Chemical Physicals (Lanzhou, China) based on the method of Yuan et al. [23]. In this experiment, 10 panelists were invited to evaluate the baked potatoes, comprising five males and five females. All of the panelists had a background in food science and received a professional training session before making evaluations. The training refers to describing, summarizing and scoring according to comprehensive sensory attributes, including color, aroma, bitterness, sweetness, texture and off-flavor, as detailed in Table S1. Samples were evaluated using a 15-point scale from 1 to 15, which corresponded to the following general descriptions that apply to each of the attributes: 1–5: poor, strong defects and dislike, 6–10: medium, no defects and like, 11–15: optimal, perfect and strong like. To ensure the authenticity and accuracy of sensory evaluation, the panelists were asked to taste the samples separately with no discussion, and descriptive analysis tests of each sample were conducted three times. The participants were also required to rest and clean their palates with water before eating the next new sample to restore their senses.

All of the participants provided written informed consent prior to study commencement, with explicit disclosure of voluntary participation rights. In the process of this research, the appropriate protocols for protecting the rights and privacy of all participants were utilized, e.g., no coercion to participate, full disclosure of study requirements and risks, written or verbal consent of participants, no release of participant data without their knowledge, and the ability to withdraw from the study at any time.

### 2.4. HS-SPME-GC-MS Analysis of Volatile Compounds of Baked Potatoes

The volatile compound determination of baked potatoes was carried out according to the methods described by Zhang et al., with slight modifications [24]. Headspace solid-phase microextraction (HS-SPME) was employed to extract the volatile compounds. Firstly, three grams of baked potato sample together with 5 mL saturated NaCl was added to a 20 mL headspace vial, followed by immediate sealing of the vial using a crimp type cap and a silicone septum (Chengdu Kelin Analytical Technology Co., Ltd., Chengdu, China). After that, the prepared sample was equilibrated in a water bath with a magnetic stirrer (800 rpm) at 40 °C for 40 min, then the volatile compounds were extracted using a SPME-arrow fiber coated with divinylbenzene/carbon wide range/polydimethylsiloxane (DVB/CAR/PDMS) (Supelco, Bellefonte, PA, USA) for 30 min under the same temperature conditions.

GC-MS (GC-MS-QP2010 Ultra) (Shimadzu, Kyoto, Japan) equipment, including a 1600 gas chromatograph system, was employed in this study. The volatile components were separated using an INNOWAX column (60 m × 250 μm × 0.25 μm) (Agilent Technologies, Santa Clara, CA, USA) at a flow rate of 1.0 mL min^−1^. The oven temperature program was set as follows: an initial 50 °C for 2 min, then the temperature was increased to 200 °C at a rate of 10 °C min^−1^ and held for 1 min. Next, the temperature was ramped up to 250 °C at a rate of 2 °C min^−1^ and held for 2 min, with solvent delay for 3 min. The ion source was electron ionization with 70 eV ionization energy, and the temperature was set at 230 °C. The ions from 35 m/z (mass/charge ratio) to 450 m/z in a full scan mode were determined. The volatile compounds of samples were characterized on the basis of matching the mass spectra and retention index (RI) according to the National Institute of Standards and Technology (NIST17) database. Various compounds with a matching degree greater than 80% were selected and maintained. The semi-quantitative analysis of volatiles was based on the normalizing of the peak areas of compounds and expressed as relative abundance (%).

### 2.5. ROAV Analysis

The key volatile compounds in the baked samples with different maturities were screened and determined using the ROAV method, which was calculated by the following equation [25]:

ROAV ≈ (100 × Ci × Tmax)/(Cmax × Ti),

where Ci represents the relative content (%) of different compounds, Cmax represents the relative content (%) of the compound contributing the most to the overall flavor, Ti is the odor threshold of each VOC in water, and Tamx is the odor threshold of the correspondent compound contributing the most to the overall flavor.

### 2.6. Molecular Docking

Molecular docking analysis was performed on the selected key volatile flavor compounds. Small molecular ligands (2-ethyl-3, 5-dimethylpyrazine (Compound CID: 26334) and 2,6-diethylpyrazine (Compound CID: 83101)) were sourced from PubChem (https://pubchem.ncbi.nlm.nih.gov/) and converted to PDB format, and the 3D structural models of olfactory receptors OR5K1 (UniProt ID: Q8NHB7) were retrieved from UniProt (https://www.uniprot.org/). Subsequently, molecular docking was performed using SeeSAR 15.0.0 and the docking engine and binding affinities were then calculated. The results were visualized using both 2D and 3D perspectives.

### 2.7. Statistical Analysis of Data

All the experiments were performed in triplicate. The final results were presented as mean ± standard deviation (SD) and visualized by Origin 2021 software (OriginLab, Northampton, MA, USA). SPSS v26.0 software (SPSS Science, Chicago, IL, USA) was used for one-way analysis of variance followed by Duncan’s multiple range test. Principal component analysis (PCA), orthogonal partial least squares-discriminant analysis (OPLS-DA) models, as well as the compounds with variable importance for the projection (VIP) calculation were measured by SIMCA 14.1 software (Umetrics, Malmö, Sweden). TBtools-II (South China Agricultural University, Guangzhou, China) was used for the clustering heat map.

## 3. Results and Discussion

### 3.1. Appearance and Flavor Analysis Among Six Cultivars with Different Maturities

Fresh and baked potatoes exhibit significant differences in color, texture, and flavor. The cut surface of fresh potatoes typically displays a yellow or white color, with a firm texture and minimal distinctive flavor. In contrast, as the heating temperature rises to 200 °C, baked potatoes undergo a series of thermal reactions that transform their surface color into a caramelized brown; meanwhile, their internal texture gradually softens, accompanied by the generation of a rich, roasted aroma.

**Figure 1:**
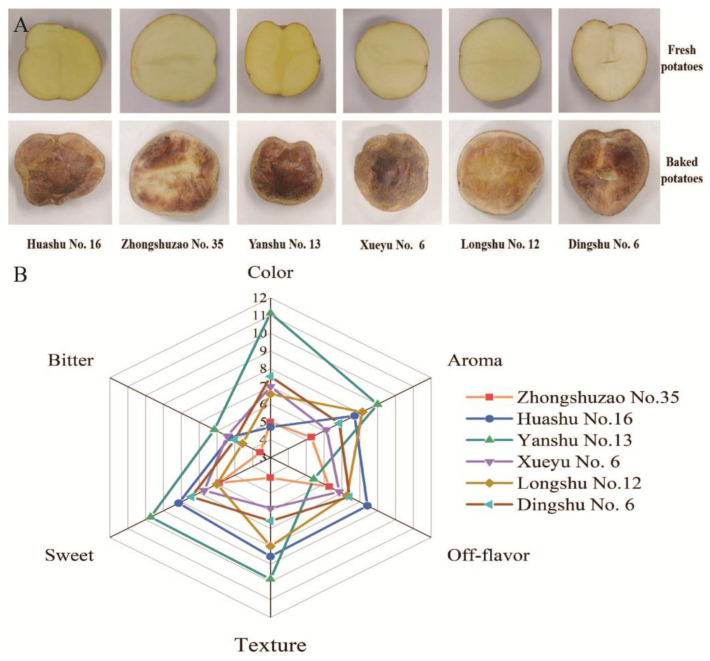
The appearance (**A**) and sensory evaluation scores (**B**) of baked potatoes from early maturing, middle-maturing and late-maturing varieties.

Among all cultivars, the texture, color, aroma, and off-flavors of potato varieties with different maturities through sensory evaluation were analyzed. The radar chart results indicate that Yan Shu No. 13 performed best in terms of texture, color, and aroma with the lowest off-flavor score (Figure 1), indicating that it was the most appealing and attractive variety for baking. In addition, there were no obvious differences in sensory characteristics for other cultivars.

The Maillard reaction, a non-enzymatic browning process between reducing sugars and amino acids, generates melanoidins and aromatic compounds that contribute to a golden-brown skin and roasted flavor [26,27]. The caramelization reaction at high temperatures leads to sugar dehydration and recombination, forming a sweet caramel flavor [28]. Starch gelatinization softens the interior, while partial starch degradation yields sugars that further fuel Maillard and caramelization reactions [29]. Additionally, the small amounts of lipids present in potato tubers are involved in the formation of aldehydes and ketones, which mainly contribute to fruity, floral, and grassy notes [30].

### 3.2. Starch and Sugar Content Analysis Among Six Cultivars with Different Maturities

**Figure 2:**
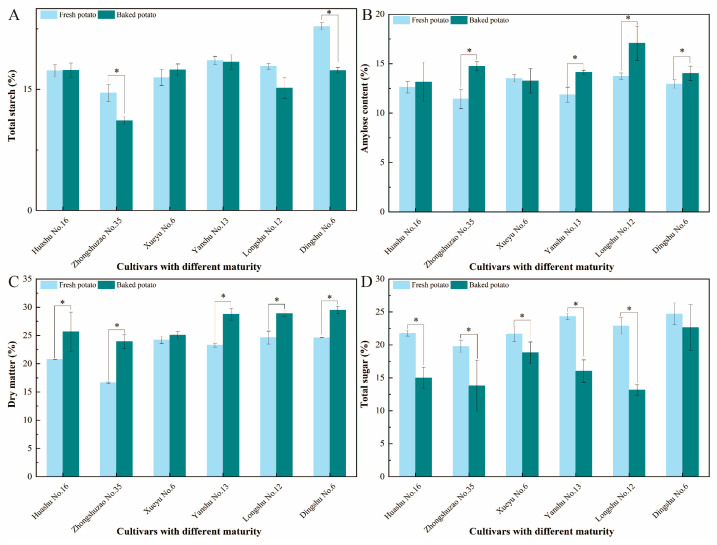
The total starch (**A**), amylose (**B**), dry matter (**C**), and total sugar (**D**) content of six cultivars with different maturity (early maturing: Huashu No. 16 and “Zhongzaoshu No. 35”; middle-maturing: ‘Xueyu No. 6’ and ‘Yanshu No. 13’; late-maturing: Longshu No. 16 and ‘Dingshu No. 6’). Asterisks indicate significant differences between fresh and baked groups (*p* < 0.05).

Starch and sugars are essential macronutrients in tuber crops, serving as major energy reserves and significantly influencing the texture, sweetness, and aroma of processed foods [31]. Upon heating, starch granules undergo gelatinization—a process involving water absorption, granule swelling, birefringence loss, and amylose leaching, which also increases viscosity [32,33]. Additionally, during the high-temperature heating process, a small fraction of starch is hydrolyzed into monosaccharides or disaccharides by α-amylase and β-amylase [34]. In this study, the total starch content in all varieties with different maturities remained largely unchanged after baking, maintaining between 17.3% and 18.3% of fresh weight, except for Zhongshuzao 35 and ‘Longshu No. 12’ (Figure 2A). This is similar to a study of cooking methods on the nutritional and physical properties of potatoes, which indicated that baking potatoes using a hot-air oven only slightly decreased the starch content [35]. However, baking significantly increased amylose content, particularly in ‘Longshu No. 12’ (Figure 2B). This likely results from starch granule disruption and amylopectin breakdown, which releases and reforms shorter amylose chains during heating [10,36].

Dry matter content increased in all cultivars after baking (25–29%) due to moisture loss (Figure 2C). Late-maturing varieties had higher dry matter, likely due to longer growth cycles promoting photosynthesis and nutrient accumulation. Fresh late-maturing varieties such as ‘Yanshu No. 13’, ‘Longshu No. 12’ and ‘Dingshu No. 6’ had the highest initial sugar content. However, the total sugar content of all the cultivars decreased significantly after baking, with ‘Dingshu No. 6’ retaining the highest sugar level (Figure 2D). Similarly, free sugars in baked and boiled sweet potatoes were also reported to decrease [37]. Sugar loss is mainly attributed to the Maillard reaction and sucrose hydrolysis during baking [38].

### 3.3. Volatile Compound Identification in Baked Potatoes with Different Maturities

**Figure 3:**
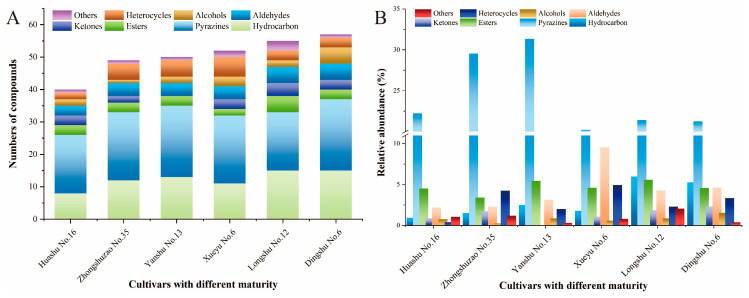
Identified volatile compounds in baked potatoes of different maturities. (**A**) Bar chart of the volatile compound numbers in different categories; (**B**) relative abundance of volatile compounds in different categories.

Baking is a traditional and common cooking method, by which food flavor can be efficiently enhanced. During baking, a series of thermal reactions between carbonyl compounds and amino acids generates complex volatile compounds, such as aldehyde, alcohols, pyrazines, etc., which impart sweet, caramel, floral and fruity aromas to the baked products [8]. In this study, the volatile compounds of baked potatoes with different maturities were analyzed using HS-SPME-GC-MS (Figure 3), and a total of 99 volatile compounds were identified from baked potato samples (Table 1). As shown in Figure 3A, the identified volatile compounds were classified into eight categories: heterocyclics (9), alcohols (8), aldehydes (10), ketones (11), esters (5), pyrazines (37), hydrocarbons (15) and others (4). Notably, late-maturing cultivars ‘Longshu No. 12’ (47 compounds) and ‘Dingshu No. 6’ (51 compounds) had the highest number of volatiles, followed by mid-maturing ‘Yanshu No. 13’ (44) and ‘Xueyu No. 6’ (46), and finally by early-maturing Huashu No. 16 (35) and ‘Zhongshuzao No. 35’ (42) (Figure 3B). This result indicates that maturity significantly influences the profile of flavor compounds in baked potatoes, with late-maturing cultivars exhibiting a greater diversity of compounds than early-maturing ones.

As is well known, pyrazines are the dominant volatile compound in baked potato [9]. Among all compounds detected across three maturity groups, pyrazines not only exhibited the greatest diversity (Figure 3A), but also displayed the highest relative abundance (Figure 3B). Therefore, abundant kinds and higher levels of pyrazine synergistically contribute to the appealing aroma of baked potatoes. However, early and middle-maturing cultivars exhibited a higher pyrazine content than late-maturing cultivars. This difference probably resulted from the levels of reducing sugars, amino acids, and moisture in the tubers at harvest [26]. ‘Zhongshuzao No. 35’ and ‘Yanshu No. 13’ had the highest proportions of pyrazines (~30%), while other cultivars were around 20%, thus resulting in their higher scores during sensory evaluation (Figure 1B).

### 3.4. Key Volatile Compounds in the Baked Potatoes

The flavor of processed potato results from a complex interplay between volatile and non-volatile compounds formed through various thermal reactions. These reactions generate a diverse array of aroma-active volatile compounds responsible for characteristic roasted, nutty, earthy, and buttery notes in baked potatoes. The types and concentrations of these volatiles determine the final sensory quality and consumer acceptance of baked potato products.

#### 3.4.1. Hydrocarbons

Hydrocarbons were the second most abundant compounds identified in this study, following pyrazines, which are primarily generated from the scission of fatty acid alkoxy radicals during heating [39]. A total of 15 hydrocarbons were identified across all samples. Results indicate that late-maturing varieties are more abundant in hydrocarbons, including decane, undecane, and dodecane (Table 1).

#### 3.4.2. Ketones

Ketones predominantly originate from lipid degradation and are recognized contributors to fatty aroma characteristics in cooked potatoes. This research identified 11 ketones including 2-hexanone, 4-methyl-2-pentanone, 2,3-heptanedione, 4,4-dimethyl-2-pentanone, 2-heptanone, 2-methyl-3-octanone, 1-octen-3-one, etc., suggesting that baking motivated the production of these ketone molecules. Specifically, 1-octen-3-one has been reported to contribute to mushroom-like odors in baked potatoes [8].

#### 3.4.3. Aldehydes

Aldehydes, primarily derived from lipid oxidation, are major aromatic-active components in boiled, baked or fried potatoes. They are associated with a wide range of flavor notes including “fatty”, “fruity”, “green”, “earthy”, and “potato-like” [40]. Typical aldehydes in baked potatoes include pentanal, hexanal, heptaldehyde, octanal, methional, furfural, benzaldehyde, 5-methyl-2-furaldehyde, benzeneacetaldehyde and 4-methyl-2-phenyl-2-pentenal (Table 1). Among these aldehydes, some contribute pleasant aromas, whilst some impart off-flavors, to the potato. For example, pentanal and hexanal give the potato “green”, “grassy”, and “almond” aromas [41], whereas octanal was described to endow potatoes with a “fatty and citrus-like” flavor [39]. Methional, a kind of sulfur-containing compound, is often identified as a key component in boiled potatoes, also found in baked potatoes, and imparts a characteristic “potato-like” odor [9,42].

#### 3.4.4. Alcohols

The formation of alcoholic compounds primarily originates from the oxidation of unsaturated fatty acids (such as linoleic acid). The resulting hydroperoxides are decomposed into aldehydes, which are further reduced to alcohols. Additionally, during Strecker degradation, amino acids (e.g., leucine, valine) react with dicarbonyl compounds to form aldehydes, which are also subsequently reduced into alcohols. These compounds impart fruity, floral, or mushroom-like aromas to food [43]. In this study, eight alcohols, namely 1-octen-3-ol, 3-methyl-1-butanol, 2,4-dimethyl-1-heptanol, 1-pentanol, leaf alcohol, 2,5-dimethyl-2,5-hexanediol, geraniol and 2-furanmethanol, were identified in different samples (Table 1). Among them, 1-octen-3-ol commonly existed in the boiled or baked potatoes, contributing to a “mushroom” flavor, and 1-pentanol provided the cooked potatoes with a burnt, unpleasant, fatty, and pungent odor [8].

#### 3.4.5. Esters

Esters are a class of compounds formed either through direct esterification between carboxylic acids and alcohols under heating conditions, or through reactions between intermediates of the Maillard reaction (e.g., hydroxyketones and aldehydes) and alcohols, leading to the production of esters with roasted aromas. These ester compounds impart fruity and floral notes to cooked foods, providing consumers with pleasant and appealing fragrances. A total of five esters were detected in our study, consisting of ethyl acetate, ethyl valerate, ethyl hexanoate, ethyl caprylate and 2-furanmethanol acetate (Table 1). Ethyl acetate and ethyl hexanoate were identified in all early, middle- and late-maturing cultivars. However, ethyl caprylate and 2-furanmethanol acetate only existed in the late-maturing cultivar ‘Longshu No. 12’.

#### 3.4.6. Heterocyclics

A series of non-enzymatic browning reactions, namely the Maillard reaction, is responsible for generating heterocyclic compounds including pyrazines, pyrroles, pyridines, furans, thiazoles and oxazoles [44]. These compounds are more readily formed at temperatures above 180 °C, which helps enhance the flavor quality of food products. However, it is important to note that excessive heating can also impart undesirable off-flavors.

#### 3.4.7. Pyrazines

Pyrazines, a subclass of nitrogen-containing heterocyclic volatile compounds, provide foods with distinctive and appealing sensory attributes including baked, roasted, and nutty flavors [45]. In this study, a total of 37 pyrazines were identified in the baked potatoes. Typical compounds such as 2-methylpyrazine, ethylpyrazine, 2, 3-dimethylpyrazine, 2-ethyl-3-methylpyrazine, 2-isobutyl-3-methylpyrazine, 5-isobutyl-2, 3-dimethylpyrazine and 2-isobutyl-3,5,6-trimethylpyrazine were common to all cultivars (Table 1). The relative abundance ranged from 0.1% to 3.3%, with compounds like 2, 6-dimethyl-3-ethylpyrazine, 2, 5-dimethyl-3-ethylpyrazine, 2-ethyl-3-methylpyrazine, and 2,5-dimethyl-3-propylpyrazine exhibiting higher proportions. These findings are consistent with previous GC-MS analyses of baked potato volatiles [9,46].

### 3.5. Analysis of Volatile Variations in Baked Samples from Different Maturities

**Figure 4:**
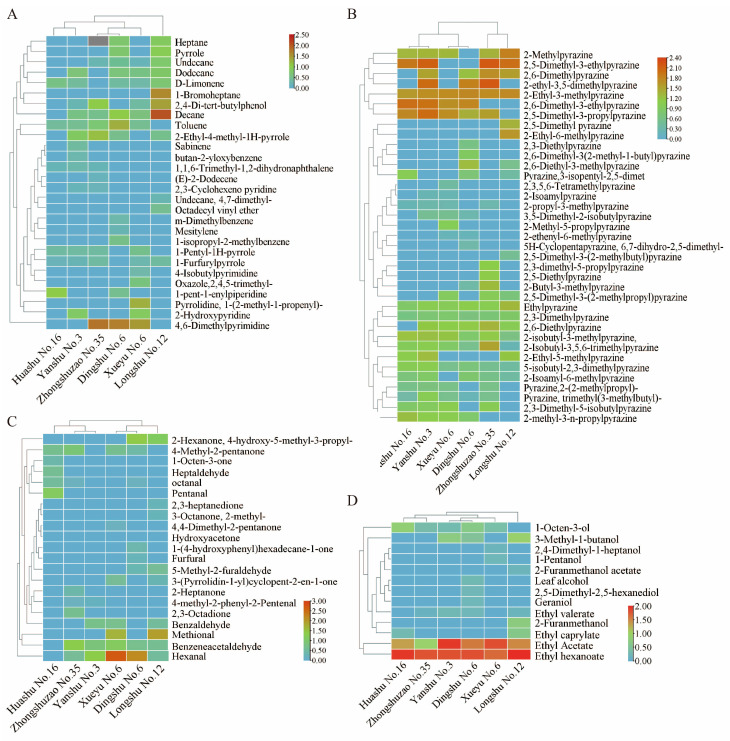
Heat maps of the different volatile compounds—(**A**) hydrocarbons and heterocyclics, except for pyrazines; (**B**) pyrazines; (**C**) aldehydes and ketones; (**D**) alcohols and esters—in baked potatoes in early maturing (‘Zhongzaoshu No. 35’ and ‘Huashu No. 16’), middle-maturing (‘Yanshu No. 13’ and ‘Xueyu No. 6’) and late-maturing (‘Longshu No. 12’ and ‘Dingshu No. 6’) cultivars. The color indicates the relative abundance of compounds; the light blue and yellowish-brown indicate the low and high peak area, respectively.

Volatile profiles reflect flavor characteristics and play a key role in the sensory differentiation of baked potato products [2]. To investigate the underlying differences in flavor characteristics among cultivars of early, middle-, and late-maturing potatoes (Table S2), key volatile compounds were further visualized using a clustering heatmap, which allows comprehensive visualization of the relative abundance of known volatile compounds. The heat maps of the hydrocarbons and heterocyclics except for pyrazines (A), pyrazines (B), alcohols and esters (C), and aldehydes and ketones (D) are shown in Figure 4. It was observed that more hydrocarbons showing relatively higher levels appeared in the late-maturing cultivars, particularly ‘Longshu No. 12’, including decane, heptane, undecane, dodecane and 1-bromoheptane (Figure 4A). Heterocyclics are generated from the pyrolysis of organic substances at temperatures above 180–200 °C, leading to a “gasoline-like odor” being imparted to the food and creating an unpleasant olfactory sensation.

As one of the typical class of heterocyclics, pyrazines were found to be the dominant volatile compounds, as previously reported [9,42]. Heatmap analysis revealed distinct pyrazine profiles for each maturity group (Figure 4B). Specifically, early maturing potatoes were rich in 2-methylpyrazine, 2,5-dimethyl-3-ethylpyrazine, 2-ethyl-3-methylpyrazine and 2,5-dimethyl-3-prophylpyrazine. The middle-maturing cultivars displayed significant levels of 2-methylpyrazine, 2-ethyl-3-methylpyrazine, 2,5-dimethyl-3-prophylpyrazine and 2,6-dimethyl-3-ethylpyrazine. Late-maturing potatoes contained elevated levels of 2-methylpyrazine, 2,5-dimethyl-3-ehtylpyrazine and 2-ethyl-3-methylpyrazine. Different pyrazines have both positive and negative roles in imparting the final flavor to baked potatoes. Part of them gives the cooked products a pleasant flavor, such as hazelnut, caramel, baked, roasted or coffee, while another part endows the products with a rubbery, bitter, musty or astringent taste [47].

Observing Figure 4C, late-maturing cultivars exhibited higher concentrations of 2-hexanone, hexanal and methional. Among them, hexanal gave the processed potatoes a grassy and rancid smell as a decomposition product of linoleic acid, whereas methional is an important component imparting an aroma of “cooked potato”. In the middle-maturing cultivars, significantly different compounds included hexanal and benzeneacetaldehyde. Generally, aldehydes typically exhibit low odor thresholds but contribute to green, citrus, or fatty aromas [48]. These two aldehydes made the middle-maturing potatoes possess fatty and floral aromas. However, the typical volatiles in early maturing potatoes are 4-methyl-2-pentanone and pentanal, which are closely related to fatty aromas in the baked samples. Regarding alcohols and esters, two kinds of esters, namely ethyl acetate and ethyl hexanoate, were identified across all of three maturities, suggesting that they were commonly responsible for the generation of a green apple flavor and pineapple smell, respectively, in the baked potatoes. In addition, other alcohol and ester compounds like 3-methyl-1-butanol, 2-furanmethanol and ethyl caprylate displayed higher levels in late-maturing cultivars, which not only endowed the late-maturing varieties with a certain roasted flavor, but also imparted a unique brandy-like fragrance to them (Figure 4D).

This study demonstrates that maturity is a significant factor influencing the differences in formation of the processed flavor in potatoes. Moreover, these differences may primarily stem from the accumulation of flavor precursors (such as fatty acids, amino acids, and sugars) in potato varieties of different maturity groups. During processing, these precursors are converted into characteristic flavor compounds through thermal reactions (such as the Maillard reaction, lipid oxidation, and Strecker degradation), thereby forming the unique flavor profiles observed in varieties with different maturities.

### 3.6. Key Volatiles Identification by Multivariate Statistical Analysis

**Figure 5:**
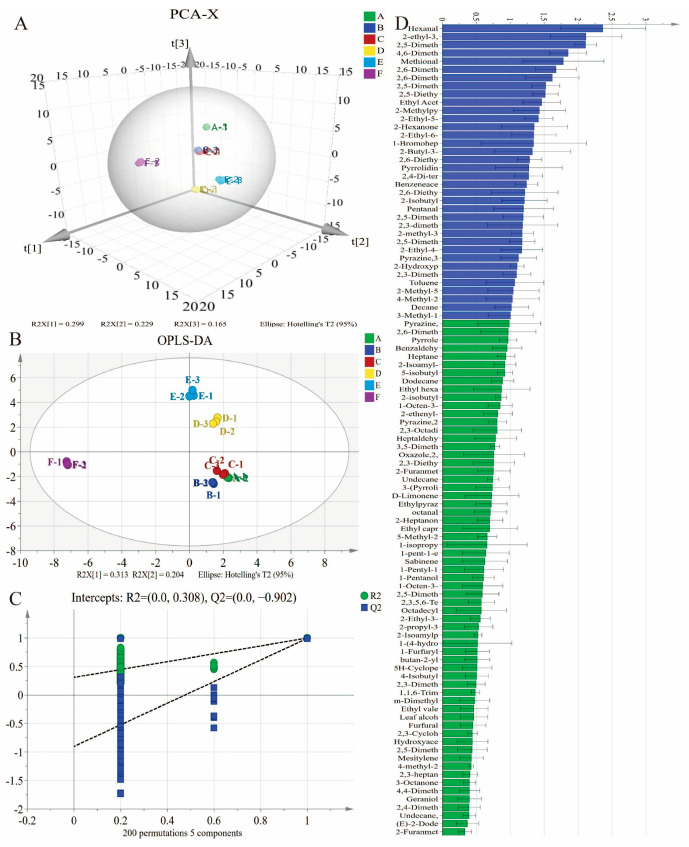
Principal component analysis (PCA) (**A**), orthogonal partial least squares-discriminant analysis (OPLS-DA) (**B**), permutation test under 200 times of OPLS-DA (**C**), and variable importance for the projection (VIP) values (**D**) of identified volatile compounds in baked potatoes. In (**A**,**B**), different color patches represent different varieties. From A to F in sequence are: ‘Zhongzaoshu No. 35’ and Huashu No. 16 (early maturing), ‘Yanshu No. 13’ and ‘Xueyu No. 6’ (middle-maturing), and ‘Longshu No. 12’ and ‘Dingshu No. 6’ (late-maturing).

The 99 identified volatile compounds among early, middle- and late-maturing cultivars were further analyzed using multivariate statistical analysis. The results of them are presented in Figure 5. PCA is usually employed to reduce data dimensionality and assess correlations among multiple variables [49]. The PCA results reveal that the cumulative variance contribution rate of the three principal components accounted for 68.3%, indicating effective statistics with comprehensive information and clear separation among groups (Figure 5A). Different color clusters show the distribution of the volatile substances of baked potatoes in six cultivars, and the spatial distances between groups reflect their differences. It was observed that the early maturing and middle-maturing cultivars, including “Zhongzaoshu No. 35”, “Huashu No. 16”, and ‘Yanshu No. 13’, showed relative proximity. This result suggests the similarities in volatile characteristics across these three groups. In contrast, ‘Longshu No. 12’ and ‘Dingshu No. 6’ are positioned at a certain distance, indicating the disparities in volatile characteristics in late-maturing cultivars. Specifically, the distributions in the quadrants show that the hydrocarbons and pyrazines are mainly responsible for the volatiles in ‘Longshu No. 12’, whereas pyrazines and esters might be accountable for the difference in ‘Dingshu No. 6’.

The OPLS-DA model, as a supervised algorithm, is always used to predict or classify the samples. As shown in Figure 5B, the values of R^2^X, R^2^Y, and Q^2^ are 0.97, 0.998 and 0.994, exhibiting high goodness-of-fit and predictive performance, confirming the model’s reliability in distinguishing samples. Permutation testing shown in Figure 5C further confirmed the model’s validity, with intercept values of R^2^ = (0.0, 0.308) and Q^2^ = (0.0, −0.902), where the negative Q^2^ indicates that the model is not overfitted and the model verification is effective. Furthermore, key volatile aroma compounds were screened out based on *p* < 0.05 and variable importance for the projection (VIP) value > 1, which was considered to play a critical role in differentiation [50]. As depicted in Figure 5D, 36 volatile compounds with VIP > 1 were screened as key variables to distinguish samples from three maturities, including three hydrocarbons, 20 pyrazines, one ester, two ketones, four aldehydes, one alcohol, four heterocyclics and one phenol. To further pinpoint key contributors, a relative odor activity value (ROAV) > 1.0 was considered a potent contributor to overall aroma [51]. Ultimately, by integrating VIP > 1 and ROAV > 1, 2-ethyl-3,5-dimethylpyrazine, 2,6-diethylpyrazine, ethyl acetate and benzeneacetaldehyde were identified as critical volatiles across the cultivars. This result further confirmed the dominant role of pyrazines in contributing roasted, nutty, and potato-like aromas in baked potatoes, while other volatiles such as ethyl acetate and benzeneacetaldehyde likely modulate the aroma by introducing fruity and floral notes.

### 3.7. Analysis of Molecular Docking of the Odorants and Olfactory Receptor

**Figure 6:**
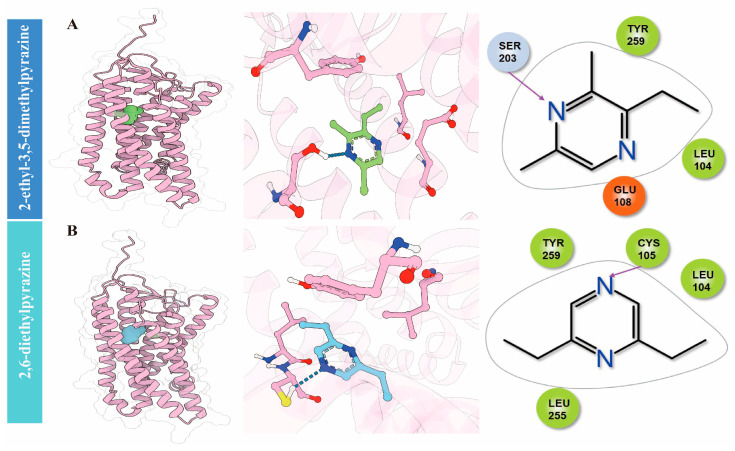
The 3D structures, binding site close-ups, and 2D interaction diagrams of OR5K1-2-ethyl-3,5-dimethylpyrazine (**A**) and OR5K1-2,6-diethylpyrazine (**B**). For the 3D structure in left, the pink represented the OR5K1, while blue and green indicated two different pyrazines. In the 2D interaction detail diagram, dashed lines represented hydrogen bonds.

Molecular docking is a computational technique used to predict the interactions between odorants in foods and olfactory receptors in the human body, providing insights into the perception and reception of flavors [52]. As is well known, pyrazine is considered the most representative aroma in roasted foods, and it has been reported that OR5K1 is a specialized olfactory receptor for detecting pyrazine-based key food odors [53]. Therefore, molecular docking was applied in this study to explore interactions between two key pyrazines (ROAV > 1) and OR5K1 by evaluating the docking scores and binding modes. Results showed that both ligands are capable of stably embedding within the OR5K1 channel and forming a favorable interaction network with the polar and hydrophobic residues surrounding the binding site. Among them, 2-ethyl-3,5-dimethylpyrazine exhibits a predicted affinity range of approximately 41 nM to 4.161 µM, suggesting a stronger binding ability, whereas 2,6-diethylpyrazine shows a predicted affinity range of 277.501 µM to 27.571 mM, indicating a relatively weaker binding capacity (Figure 6). Previous studies demonstrated that the activation of OR5K1 by odorants with low EC50 mainly included pyrazines, suggesting that these kinds of compounds were effective at activating the receptor [53].

Further analysis of the binding modes reveals that in the 2-ethyl-3,5-dimethylpyrazine system, the ligand is tightly embedded within the channel, with its pyrazine ring at the core. A stable hydrogen bond is formed between the side-chain hydroxyl group of SER203 and the nitrogen atom on the pyrazine ring, providing a key polar contribution for anchoring the ligand-binding orientation. Meanwhile, residues such as TYR259 and LEU104 form hydrophobic and van der Waals contacts around the ligand, further stabilizing its binding conformation. In the 2,6-diethylpyrazine system, the two ethyl groups of the ligand extend into the hydrophobic regions on either side of the channel. A hydrogen bond is formed between the nitrogen atom on the pyrazine ring and the sulfhydryl group of CYS105, serving as the primary polar anchor for ligand binding. Additionally, residues including TYR259, LEU104, and LEU255 maintain close contact with the ethyl groups and pyrazine ring of 2,6-diethylpyrazine through hydrophobic interactions, contributing to the stabilization of its binding conformation. These findings suggest that the key aroma compounds can bind effectively to the corresponding receptors through stable hydrogen bonds and hydrophobic and van der Waals interactions, providing molecular-level support for their role in triggering pleasant sensory responses. It should be noted that the above binding modes are derived from molecular docking predictions, and the exact interaction patterns require further experimental validation.

## 4. Conclusions

Differences in the proximate composition and volatile flavor compounds present in baked potatoes with different maturities were analyzed in this study. After baking, all cultivars exhibited a significant increase in dry matter and amylose contents and a decrease in total sugars. HS-SPME-GC-MS identified a total of 99 volatile compounds including nine heterocyclics, eight alcohols, 10 aldehydes, 11 ketones, five esters, 37 pyrazines, 15 hydrocarbons and four others. Pyrazines were the most dominant class, with ‘Yanshu No. 13’ exhibiting the highest relative abundance, which lead to its superior aroma score in the sensory evaluation. Notably, the number and diversity of volatile compounds increased with maturity, suggesting that late-maturing cultivars possess a more complex aroma profile. Further, the combined analysis of multivariate statistical analysis and ROAV identified four key differential volatiles (2-ethyl-3,5-dimethylpyrazine, 2,6-diethylpyrazine, ethyl acetate and benzeneacetaldehyde) as major contributors to the characteristic aroma of baked potatoes. Moreover, the molecular docking results demonstrated the interaction mode between two key pyrazines and olfactory receptor OR5K1 mainly via hydrogen bonds. The findings in this project provide theoretical support for interpreting the effect of potato maturity on the final flavor profile of baked potatoes as well as preliminarily exploring the aroma perception mode of baked foods.

## Figures and Tables

**Table 1:** Key compounds identified in baked potatoes of different maturities.

Compounds	Formula	MW	CAS #	RT	RI
Heptane	C_7_H_16_	100	142-82-5	3.965	717
Decane	C_10_H_22_	142	124-18-5	7.448	1015
Toluene	C_7_H_8_	92	108-88-3	8.534	794
Undecane	C_11_H_24_	156	1120-21-4	8.868	1115
Sabinene	C_10_H_16_	136	3387-41-5	9.716	897
m-Dimethylbenzene	C_8_H_10_	106	108-38-3	10.204	907
Dodecane	C_12_H_26_	170	112-40-3	10.554	1214
Undecane, 4,7-dimethyl-	C_13_H_28_	184	17301-32-5	10.828	1185
D-Limonene	C_10_H_16_	136	5989-27-5	11.067	1018
(E)-2-Dodecene	C_12_H_24_	168	7206-13-5	11.258	1222
1-isopropyl-2-methylbenzene	C_10_H_14_	134	527-84-4	12.431	1042
Mesitylene	C_9_H_12_	120	108-67-8	12.616	1020
1-Bromoheptane	C_7_H_15_Br	178	629-04-9	15.181	1013
Butan-2-yloxybenzene	C_10_H_14_O	150	10574-17-1	16.746	1104
1,1,6-Trimethyl-1,2-dihydronaphthalene	C_13_H_16_	172	30364-38-6	19.537	1396
2-Methylpyrazine	C_5_H_6_N_2_	94	109-08-0	12.379	781
2,5-Dimethyl pyrazine	C_6_H_8_N_2_	108	123-32-0	13.223	894
2,6-Dimethylpyrazine	C_6_H_8_N_2_	108	108-50-9	13.308	894
Ethylpyrazine	C_6_H_8_N_2_	108	13925-00-3	13.425	881
2,3-Dimethylpyrazine	C_6_H_8_N_2_	108	5910-89-4	13.619	894
2-Ethyl-6-methylpyrazine	C_7_H_10_N_2_	122	13925-03-6	14.13	994
2-Ethyl-5-methylpyrazine	C_7_H_10_N_2_	122	13360-64-0	14.258	994
2-Ethyl-3-methylpyrazine	C_7_H_10_N_2_	122	15707-23-0	14.42	994
2,6-Diethylpyrazine	C_8_H_12_N_2_	136	13067-27-1	14.829	1093
2,5-Dimethyl-3-ethylpyrazine	C_8_H_12_N_2_	136	13360-65-1	14.945	1107
Pyrazine,2-(2-methylpropyl)-	C_8_H_12_N_2_	136	29460-92-2	15.104	1015
2,3-Diethylpyrazine	C_8_H_12_N_2_	136	15707-24-1	15.11	1093
2-ethyl-3,5-dimethylpyrazine	C_8_H_12_N_2_	136	13925-07-0	15.193	1107
2,3,5,6-Tetramethylpyrazine	C_8_H_12_N_2_	136	1124-11-4	15.365	1121
2-propyl-3-methylpyrazine	C_8_H_12_N_2_	136	15986-80-8	15.471	1093
2,6-Dimethyl-3-ethylpyrazine	C_9_H_14_N_2_	150	13610-20-3	15.564	1142
2,6-Diethyl-3-methylpyrazine	C_9_H_14_N_2_	150	18138-05-1	15.615	1206
2-ethenyl-6-methylpyrazine	C_7_H_8_N_2_	120	13925-09-2	15.688	984
2-isobutyl-3-methylpyrazine	C_9_H_14_N_2_	150	13925-06-9	15.745	1129
2-methyl-3-n-propylpyrazine	C_9_H_14_N_2_	150	17398-16-2	15.849	1220
2,3-dimethyl-5-propylpyrazine	C_9_H_14_N_2_	150	32262-98-9	15.854	1206
2,5-Dimethyl-3-(2-methylpropyl)pyrazine	C_10_H_16_N_2_	164	32736-94-0	16.16	1242
5-isobutyl-2,3-dimethylpyrazine	C_10_H_16_N_2_	164	54410-83-2	16.25	1242
3,5-Dimethyl-2-isobutylpyrazine	C_10_H_16_N_2_	164	70303-42-3	16.405	1242
2-Isobutyl-3,5,6-trimethylpyrazine	C_11_H_18_N_2_	178	46187-37-5	16.475	1355
2,5-Diethylpyrazine	C_8_H_12_N_2_	136	13238-84-1	16.62	1093
2-Isoamylpyrazine	C_9_H_14_N_2_	150	40790-22-5	16.893	1115
2-Butyl-3-methylpyrazine	C_9_H_14_N_2_	150	15987-00-5	17.102	1193
2-Isoamyl-6-methylpyrazine	C_10_H_16_N_2_	164	91010-41-2	17.344	1228
2-Methyl-5-propylpyrazine	C_8_H_12_N_2_	136	29461-03-8	17.353	1093
2,5-Dimethyl-3-(2-methylbutyl)pyrazine	C_11_H_18_N_2_	178	72668-36-1	17.422	1341
2,5-Dimethyl-3-propylpyrazine	C_9_H_14_N_2_	150	18433-97-1	17.431	1206
2,6-Dimethyl-3(2-methyl-1-butyl)pyrazine	C_11_H_18_N_2_	178	56617-70-0	17.432	1341
2,3-Dimethyl-5-isobutylpyrazine	C_10_H_16_N_2_	164	15834-78-3	17.646	1306
Pyrazine,3-isopentyl-2,5-dimet	C_11_H_18_N_2_	178	18433-98-2	17.73	1341
Pyrazine, trimethyl(3-methylbutyl)-	C_12_H_20_N_2_	192	10132-43-1	18.021	1454
5H-Cyclopentapyrazine, 6,7-dihydro-2,5-dimethyl-	C_9_H_12_N_2_	148	38917-61-2	18.306	1208
Ethyl acetate	C_4_H_8_O_2_	88	141-78-6	5.952	586
Ethyl valerate	C_7_H_14_O_2_	130	539-82-2	9.865	884
Ethyl hexanoate	C_8_H_16_O_2_	144	123-66-0	11.501	984
Ethyl caprylate	C_10_H_20_O_2_	172	106-32-1	14.529	1183
2-Furanmethanol acetate	C_7_H_8_O_3_	140	623-17-6	16.067	1009
2-Hexanone, 4-hydroxy-5-methyl-3-propyl-	C_10_H_20_O_2_	172	61141-74-0	7.881	1185
4-Methyl-2-pentanone	C_6_H_12_O	100	108-10-1	7.896	690
2,3-heptanedione	C_7_H_12_O_2_	128	96-04-8	10.133	989
4,4-Dimethyl-2-pentanone	C_7_H_14_O	114	590-50-1	10.14	769
2-Heptanone	C_7_H_14_O	114	110-43-0	10.814	853
3-Octanone, 2-methyl-	C_9_H_18_O	142	923-28-4	11.896	988
1-Octen-3-one	C_8_H_14_O	126	4312-99-6	12.738	943
2,3-Octadione	C_8_H_14_O_2_	142	585-25-1	12.922	1088
Hydroxyacetone	C_3_H_6_O_2_	74	116-09-6	12.96	698
1-(4-hydroxyphenyl) Hexadecane-1-one	C_22_H_36_O_2_	332	2589-76-6	18.271	2641
3-(Pyrrolidin-1-yl)cyclopent-2-en-1-one	C_9_H_13_NO	151	36287-28-2	18.472	1335
Pentanal	C_5_H_10_O	86	110-62-3	7.446	707
Hexanal	C_6_H_12_O	100	66-25-1	9.096	806
Heptaldehyde	C_7_H_14_O	114	111-71-7	10.85	905
Octanal	C_8_H_16_O	128	124-13-0	12.533	1005
Methional	C_4_H_8_OS	104	3268-49-3	15.241	858
Furfural	C_5_H_4_O_2_	96	1998/1/1	15.308	831
Benzaldehyde	C_7_H_6_O	106	100-52-7	16.36	982
5-Methyl-2-furaldehyde	C_6_H_6_O_2_	110	620-02-0	16.858	920
Benzeneacetaldehyde	C_8_H_8_O	120	122-78-1	17.916	1081
4-methyl-2-phenyl-2-Pentenal	C_12_H_14_O	174	26643-91-4	22.575	1400
3-Methyl-1-butanol	C_5_H_12_O	88	123-51-3	11.004	697
2,4-Dimethyl-1-heptanol	C_9_H_20_O	144	98982-97-9	11.259	1030
1-Pentanol	C_5_H_12_O	88	71-41-0	11.68	761
Leaf alcohol	C_6_H_12_O	100	928-96-1	13.807	868
1-Octen-3-ol	C_8_H_16_O	128	3391-86-4	14.613	969
2-Furanmethanol	C_5_H_6_O_2_	98	98-00-0	17.625	885
2,5-Dimethyl-2,5-hexanediol	C_8_H_18_O_2_	146	110-03-2	18.786	1000
Geraniol	C_10_H_18_O	154	106-24-1	20.24	1228
Pyrrolidine, 1-(2-methyl-1-propenyl)-	C_8_H_15_N	125	2403-57-8	13.047	996
4,6-Dimethylpyrimidine	C_6_H_8_N_2_	108	1558-17-4	13.239	894
1-Pentyl-1H-pyrrole	C_9_H_15_N	137	699-22-9	13.933	1075
2-Hydroxypyridine	C_5_H_5_NO	95	142-08-5	15.921	847
Pyrrole	C_4_H_5_N	67	109-97-7	15.931	710
4-Isobutylpyrimidine	C_8_H_12_N_2_	136	98489-37-3	16.753	1015
2-Ethyl-4-methyl-1H-pyrrole	C_7_H_11_N	109	69687-77-0	18.525	988
1-Furfurylpyrrole	C_9_H_9_NO	147	1438-94-4	20.537	1199
2,3-Cyclohexeno pyridine	C_9_H_11_N	133	10500-57-9	21.226	1160
Oxazole,2,4,5-trimethyl-	C_6_H_9_NO	111	20662-84-4	11.021	859
Octadecyl vinyl ether	C_20_H_40_O	296	930-02-9	12.203	2075
1-pent-1-enylpiperidine	C_10_H_19_N	153	49845-25-2	13.043	1238
2,4-Di-tert-butylphenol	C_14_H_22_O	206	96-76-4	30.402	1555

Note: Chemical abstracts service. MW: molecular weight, CAS: Chemical abstract service, RI: retention index, RT: retention time.

## Data Availability

The original contributions presented in this study are included in the article/Supplementary Materials. Further inquiries can be directed to the corresponding authors.

## Supplementary Materials

The following supporting information can be downloaded at: https://www.mdpi.com/article/10.3390/foods15091468/s1, Table S1: Detailed guidelines for sensory assessment; Table S2: Relative abundance of compounds in the baked potatoes of early, middle- and late-maturing cultivars.
