# Supplementary material for: Maturity-Dependent Volatile Flavor Profiling of Baked Potatoes via HS-SPME-GC-MS, Multivariate Statistical Analysis, and Computational Modeling
Source: Foods. 2026 Apr 22;15(9):1468. doi: 10.3390/foods15091468 (PMC13163579; doi:10.3390/foods15091468)
Supplement: Supplementary file 1 [file foods-15-01468-s001.zip › foods-4240695-supplementary.pdf]

Table S1. Detailed Guidelines for Sensory Assessment

Evaluation parameters	Value	Scoring Criteria
**Color**	15	Pale white（1-5）<br>Light yellow（6-10）<br>Golden yellow（11-15）
**Aroma**<br>**（Roasted aroma）**	15	Poor roasting aroma, almost none（1-5）<br>Moderate roasting aroma（6-10）<br>Rich roasting aroma with distinct potato notes（11-15）
**Off-flavor**	15	Strong off-odors (e.g., earthy notes)（1-5）<br>Slight off-odors（6-10）<br>Completely free of off-odors（11-15）
**Texture**	15	Not mealy, hard texture（1-5）<br>Moderately mealy（6-10）<br>Excellent mealiness with granular perception（11-15）
**Bitter**	15	Bitter with poor taste（1-5）<br>Slight bitterness, acceptable（6-10）<br>No bitterness, with umami-sweet notes（11-15）
**Sweet**	15	Very faint or no sweetness（1-5）<br>Mild sweetness（6-10）<br>Pronounced sweetness, highly perceptible（11-15）

Table S2. Relative abundance of compounds in the baked potatoes of early-, middle- and late-maturing cultivars.

Compoundds	Relative abundance (%)					
	Huashu No.16	Zhongshuzao No. 35	Yanshu No. 13	Xueyu No. 6	Longshu No. 12	'Dingshu No. 6'
Heptane					0.81±0.09^{a}	0.68±0.07^{b}
Decane		0.42±0.02^{d}	0.49±0.02^{d}	0.54±0^{c}	3.09±0.05^{a}	1.07±0.01^{b}
Toluene	0.33±0.04^{c}	0.65±0^{b}	0.38±0.03^{c}	0.38±0.04^{c}		1.46±0.03^{a}
Undecane		0.18±0.01^{c}		0.14±0.02^{c}	0.7±0^{a}	0.37±0.19^{b}
Sabinene			0.31±0.07			
m-Dimethylbenzene						0.22±0.03
Dodecane			0.59±0.06^{b}	0.49±0.04^{c}	0.75±0.09^{a}	0.58±0.12^{b}
Undecane, 4,7-dimethyl-					0.18±0.02	
D-Limonene	0.48±0.11^{b}		0.33±0.05^{c}	0.24±0.01^{d}	0.63±0.33^{a}	0.3±0.03^{c}
(E)-2-Dodecene		0.12±0.01				
1-isopropyl-2-methylbenzene						0.45±0.28
Mesitylene						0.17±0
1-Bromoheptane					2.1±0.84	
butan-2-yloxybenzene			0.2±0.02			
1,1,6-Trimethyl-1,2-dihydronaphthalene	0.15±0.01^{b}	0.15±0^{b}	0.21±0.01^{a}			
2-Methylpyrazine	1.53±0.08^{b}	1.62±0.11^{b}	1.55±0.18^{b}	1.5±0.18^{b}	2.57±0.21^{a}	
2,5-Dimethyl pyrazine					1.63±0.04	
2,6-Dimethylpyrazine		2.27±0.04^{a}	1.87±0.38^{b}		1.99±0.72^{b}	1.35±0.16^{c}
Ethylpyrazine	0.79±0.01^{d}	1.04±0.02^{c}	1.06±0.01^{c}	0.99±0.03^{c}	1.51±0.09^{a}	1.11±0.02^{b}
2,3-Dimethylpyrazine	0.57±0.04^{b}	0.67±0.02^{ab}	0.7±0.02^{a}	0.77±0.04^{a}	0.81±0.06^{a}	0.59±0^{b}
2-Ethyl-6-methylpyrazine					2.09±0.02	
2-Ethyl-5-methylpyrazine	1.13±0.02^{c}		1.43±0^{a}		1.28±0.01^{b}	
2-Ethyl-3-methylpyrazine	2.05±0^{b}	2.34±0.02^{ab}	2.31±0.08^{ab}	2.35±0.11^{ab}	2.37±0.01	2.41±0.04^{a}
2,6-Diethylpyrazine		1.56±0.04^{a}	1.25±0.31^{b}	1.14±0.4^{c}	1.01±0.03^{d}	1.22±0.33^{b}
2,5-Dimethyl-3-ethylpyrazine	2.76±0.09^{d}	3.54±0.04^{a}	3.33±0.03^{b}		2.96±0.04^{c}	
Pyrazine,2-(2-methylpropyl)-	0.48±0^{b}	0.51±0.03^{a}	0.59±0.02^{a}	0.51±0.01^{a}		
2,3-Diethylpyrazine						0.53±0.01
2-ethyl-3,5-dimethylpyrazine		3.54±0.25^{a}	3.01±0.29^{b}			2.56±0.35^{c}
2,3,5,6-Tetramethylpyrazine				0.3±0.01		
2-propyl-3-methylpyrazine	0.17±0^{b}	0.21±0.02^{a}	0.22±0.02^{a}	0.21±0^{a}		
2,6-Dimethyl-3-ethylpyrazine	2.94±0.68^{a}		2.91±0.52^{a}	2.24±0.02^{c}		2.46±0.6^{b}
2,6-Diethyl-3-methylpyrazine					0.46±0.05^{b}	1.41±0.02^{a}
2-ethenyl-6-methylpyrazine				0.15±0^{a}		0.15±0^{a}
2-isobutyl-3-methylpyrazine,	1.34±0.06^{a}	1.2±0.07^{ab}	1.38±0.03^{a}	1.31±0.04^{a}	0.56±0.02^{c}	0.78±0.06^{b}
2-methyl-3-n-propylpyrazine	1.4±0.12^{a}		1.13±0.04^{b}	1.08±0.02^{b}		0.78±0.03^{c}
2,3-dimethyl-5-propylpyrazine		1.16±0.01				

2,5-Dimethyl-3-(2-methylpropyl)pyrazine		0.99±0.09^{a}		0.9±0.02^{a}	0.62±0.02^{b}	
5-isobutyl-2,3-dimethylpyrazine	1±0.01^{a}	0.43±0.04^{c}	1.09±0.07^{a}	0.46±0.01^{c}	0.33±0.02^{d}	0.64±0.05^{b}
3,5-Dimethyl-2-isobutylpyrazine			0.41±0.03^{a}	0.43±0a		0.21±0^{b}
2-Isobutyl-3,5,6-trimethylpyrazine	0.96±0.02^{c}	1.88±0.12^{a}	1.09±0.07^{b}	0.85±0.03^{d}	0.18±0.02^{f}	0.46±0.05^{e}
2,5-Diethylpyrazine		1.17±0.04				
2-Isoamylpyrazine			0.18±0^{a}	0.19±0.01^{a}		
2-Butyl-3-methylpyrazine		1.47±0.08^{a}				0.27±0^{a}
2-Isoamyl-6-methylpyrazine	0.63±0.01^{c}	0.5±0^{d}	0.83±0.01^{a}		0.32±0.06^{e}	0.71±0.02^{b}
2-Methyl-5-propylpyrazine				0.98±0.05		
2,5-Dimethyl-3-(2-methylbutyl)pyrazine					0.41±0.1	
2,5-Dimethyl-3-propylpyrazine	2.41±0.02^{b}	2±0.08^{d}	3.05±0.13^{a}	2.3±0.1^{c}		1.69±0.06^{e}
2,6-Dimethyl-3(2-methyl-1-butyl)pyrazine						0.88±0.03
2,3-Dimethyl-5-isobutylpyrazine	0.8±0.01^{b}	1±0.02^{a}	0.92±0.03^{ab}	1.1±0.08^{a}		
Pyrazine,3-isopentyl-2,5-dimet	1.02±0.01^{a}				0.32±0.03^{c}	0.65±0.02^{b}
Pyrazine, trimethyl(3-methylbutyl)-	0.28±0.01^{c}	0.46±0.05^{b}	1.02±0.21^{a}	0.46±0.07^{b}		0.16±0.01^{d}
5H-Cyclopentapyrazine, 6,7-dihydro-2,5-dimethyl-						0.25±0.03
Ethyl Acetate	1.54±0.06^{e}	0.91±0.13^{f}	2.81±0.12^{a}	2.5±0.09^{b}	1.78±0.28^{d}	1.94±0.11^{c}
Ethyl valerate		0.11±0^{a}	0.19±0.11^{a}		0.14±0.02^{a}	0.15±0^{a}
Ethyl hexanoate	2.78±0.21^{b}	2.4±0.06^{c}	2.45±0.29^{c}	2.12±0.11^{d}	3±0.4^{a}	2.48±0.46^{c}
Ethyl caprylate	0.21±0.01^{b}				0.55±0.18^{a}	
2-Furanmethanol acetate					0.13±0.02	
2-Hexanone, 4-hydroxy-5-methyl-3-propyl-					1.25±0.3^{b}	1.67±0.33^{a}
4-Methyl-2-pentanone	0.57±0.09^{b}	0.88±0.24^{a}		0.55±0.05^{b}		0.37±0.06^{c}
2,3-heptanedione					0.19±0.02	
4,4-Dimethyl-2-pentanone				0.14±0.02		
2-Heptanone		0.3±0.11				
3-Octanone, 2-methyl-					0.19±0.01	
1-Octen-3-one		0.31±0.07				
2,3-Octadione		0.56±0.11				
Hydroxyacetone						
1-(4-hydroxyphenyl)hexadecane-1-one						0.27±0.13
3-(Pyrrolidin-1-yl)cyclopent-2-en-1-one				0.49±0.01^{a}	0.23±0.04^{b}	
Pentanal	1.21±0.03					
Hexanal		0.49±0.02^{d}	1.6±0.04^{c}	5.5±0.39^{a}	0.46±0.02^{d}	3.46±0.77^{b}
Heptaldehyde	0.53±0.04					
octanal	0.45±0.03^{a}	0.14±0.02^{b}				0.14±0.01^{b}
Methional				2.21±0.16^{b}	2.8±1.18^{a}	
Furfural						0.19±0.01
Benzaldehyde			0.62±0.19^{a}	0.59±0.13^{a}	0.46±0.01^{b}	
5-Methyl-2-furaldehyde					0.49±0.04^{a}	0.26±0.17^{b}
Benzeneacetaldehyde		1.52±0.01^{a}	0.8±0.2^{c}	1.24±0.03^{b}	0.54±0.03^{d}	0.6±0.06^{d}
4-methyl-2-phenyl-2-Pentenal		0.14±0.01^{a}	0.12±0^{a}			

1-Octen-3-ol	0.77±0.02^{a}	0.26±0.03^{c}	0.23±0.06^{c}	0.29±0.03^{c}		0.59±0.02^{b}
3-Methyl-1-butanol			0.64±0.1^{b}		0.87±0.26^{a}	0.43±0.08^{c}
2,4-Dimethyl-1-heptanol				0.14±0.02		
1-Pentanol				0.16±0.01		
Leaf alcohol						0.2±0.02
2,5-Dimethyl-2,5-hexanediol						0.18±0.03
Geraniol						0.15±0.02
2-Furanmethanol					0.66±0.05	
Pyrrolidine, 1-(2-methyl-1-propenyl)-				1.45±0.23		
4,6-Dimethylpyrimidine		2.45±0.01^{a}		1.81±0.34^{c}		2.24±0.05^{b}
1-Pentyl-1H-pyrrole	0.27±0.07^{b}	0.29±0.04^{b}	0.28±0.1^{b}	0.42±0.17^{a}		
2-Hydroxypyridine			0.77±0.02^{a}	0.82±0^{a}		
Pyrrole					0.92±0.04^{a}	0.68±0.05^{b}
4-Isobutylpyrimidine				0.23±0		
2-Ethyl-4-methyl-1H-pyrrole		1.23±0.15^{a}	0.8±0.17^{b}		0.42±0.09^{c}	0.43±0.07^{c}
1-Furfurylpyrrole	0.13±0^{c}	0.27±0.09^{b}	0.16±0.01^{c}	0.2±0^{b}	0.31±0.06^{a}	
2,3-Cyclohexeno pyridine		0.13±0.02^{a}	0.14±0.02^{a}			
Oxazole,2,4,5-trimethyl-				0.53±0.16		
Octadecyl vinyl ether					0.38±0.13	
2,4-Di-tert-butylphenol		1.06±0.13^{b}	0.18±0.02^{c}	0.27±0.02^{c}	1.68±0.14^{a}	
1-pent-1-enylpiperidine	1.07±0.14^{a}					0.39±0.07^{b}

Note: Blanks means not detected.
